# Minimally invasive–percutaneous surgery – recent developments of the foot surgery techniques

**Published:** 2015

**Authors:** I Botezatu, R Marinescu, D Laptoiu

**Affiliations:** * Colentina Clinical Hospital, Bucharest Romania

**Keywords:** minimally invasive–percutaneous surgery, foot surgery techniques, osteotomies, hallux valgus treatment, intermetatarsal angle

## Abstract

Percutaneous techniques are currently more and more used in many surgical procedures on the soft tissues and bones of the foot. Practical advantages include lower complication rates and faster recovery times. Potential disadvantages are related to the need for specific equipment and extensive learning curve. One of the most frequent techniques involves a combination of chevron osteotomy of the first metatarsal with osteotomy of the first phalanx, both internally fixated. Lateral metatarsal misalignment and toe deformities can also be addressed by percutaneous treatment, with lower morbidity rates than open techniques. The most commonly performed percutaneous procedures are described, with their current indications, outcomes, and recent developments.

## Introduction

The surgical correction of deformities of the forefoot had a traditional bad reputation because of peri-operative pain, surgical imperfections, scars and risk of recurrence. Much of these complications are not related to the actual surgical techniques, but to errors in the surgical indication or execution. In front of a patient with foot problems, we have to analyze the nature of the complaints and expectations in order to propose an adapted intervention and, surgeons must learn to refuse to operate on a patient without suggestive symptoms. Since the 90’s, osteotomies of the first metatarsal were proposed by Samuel Barouk and developed in France, quickly becoming one of the most selected techniques for the correction of the hallux valgus [**1**]. During the second half of the 20th century, many surgical specialties have evolved towards minimally invasive techniques. After 2000, Mariano de Prado popularized in Europe new percutaneous techniques of foot osteotomy. Minimally invasive surgery of forefoot or percutaneous surgery may provide better outcomes for patients [**2**], because of potentially decreased postoperative morbidity (limited pain and rare wound problems because of smaller deep tissue dissection, reduction of stiffness), decreasing recovery and rehabilitation times, which finally allows ambulatory surgery and immediate full weight-bearing with a rigid flat-bottom shoe [**3**].

Percutaneous surgery is defined by being performed through the smallest possible working incision, without direct visualization of the underlying target structures, by using several specialized instruments: a mini-blade for soft tissue incision, a power rotatory burr for bony procedures and intra-operative fluoroscopy control. Bone fixation materials cannot always be utilized since an incision space is not enough but dedicated techniques are currently under development. That is why bones are held in place mostly by a solid dressing, optionally followed by small silicone blocks, for the time of bone healing. This technique is hard to practice, requiring additional learning from experts and good knowledge of classical “open” techniques. Anatomic and technical knowledge of these evolving techniques, is supported by the professional association of GRECMIP (Groupe de recherche et d’énseignement en chirurgie mini-invasive du pied) [**4**].

## Material

Many operative procedures have been described to correct the hallux valgus deformity, but today there is no consensus on the outcomes of hallux valgus treatment. In the last few decades, several techniques have been increasingly used: CHEVRON-type osteotomy (distal first metatarsal, V-shaped), SCARF osteotomy (complex, 3Dimensional, multiplanar, z-shaped), LAPIDUS arthrodesis and MTP1 (first metatarsophalangeal) arthrodesis, in combination or not with AKIN osteotomy (phalangeal), DSTP and WEIL osteotomy (second to fourth metatarsal) depending on hallux valgus severity and its impact on other anatomical structures of the foot [**5**].

Percutaneous surgery is increasing in popularity for hallux valgus correction and this is an interesting area of development. According to the latest papers, the most commonly performed percutaneous techniques are MICA (Chevron+Akin), DMMO, Correction of Lateral Metatarsal Malalignment and Toes Deformities, Corection of Tailor’s Bunnionete, MTP1 Arthrodesis, TMT1 Arthrodesis [**5**,**6**]. The success of such precision operations crucially depends on the selection of the best procedure for the individual deformity. 

One of the most used classifications in the current orthopedic practice is the Coughlin classification with MTO being the metatarsophalangeal angle, IM, the intermetatarsal angle and sesamoid position [**7**]:

• Mild (MTP angle <20°, IM angle <11°, lateral sesamoid subluxation <50%)

• Moderate (MTP angle 20-40°, IM angle <16°, lateral sesamoid subluxation 50-70%)

• Severe (MTP angle >40°, IM angle >16°, lateral sesamoid subluxation >75%)

A first observation is that classifications from literature do not provide enough knowledge support to decide the treatment protocol. There are different types of hallux valgus, different types of operations and a solid selection of the technique, based on clinical elements and complete imaging investigations (X-rays, MRI, CT scans when needed), are needed. There is not a simple and practical way to define the best procedure needed (too many clinical aspects need to be considered in each case). The ideal surgical procedure should be able to concomitantly correct HV (hallux valgus) and IMA (intermetatarsal angle), restore joint congruity, eliminate pain, and preserve range of motion [**8**].

Exostosectomy, lateral metatarso-phalangeal arthrolysis, and osteotomy of the first phalanx are used in almost all procedures and can be performed in all cases of HV. We started to correct mild to moderate HV (up to 18°) by using the Minimally Invasive Chevron-Akin (MICA). The technique involves a percutaneous chevron-type osteotomy at the level of the distal diaphyseal-metaphyseal junction of the first metatarsal and a percutaneous Akin-type osteotomy of the hallux P1, both of which are internally fixed with compression screws (2 for Chevron and 1 for Akin) all combined with a percutaneous distal soft tissue release [**9**]. All the cases we operated required fixation with screw to provide stability and compression. The key technique point is that one of the screws (the proximal one) of the Chevron should provide tricortical fixation. The Chevron osteotomy is a V shaped cut (almost to 90°) extraarticular and distal metatarsal osteotomy and the bone is displaced laterally by one third to a half of its width, thus correcting the intermetatarsal angle.

Akin osteotomy is a basal closing wedge osteotomy of the hallux first phalanx, designed to realign the forces over the tendon of extensor hallucis longus, thus straightening the first ray [**10**].

**Fig. 1:**
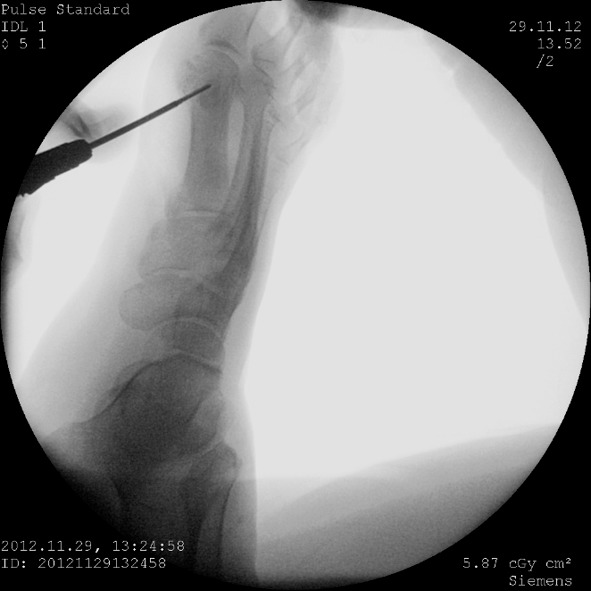
Intraoperative aspect under fluoroscopic control performing first metatarsal osteotomy

Metatarsalgia (pain in the forefoot under the metatarsal heads), is often related to a first ray deficiency (the so called “transfer metatarsalgia”). In our practice, surgical treatment is indicated when other measures fail, guided by clinical observations and preoperative radiographs. Realignment and reconstruction of the first ray is one important issue to consider in treating transfer metatarsalgia. The goal of current techniques is getting a shortening of metatarsal bones to improve forefoot-loading pattern. WEIL osteotomy remains the most popular, but Distal Minimally Invasive Metatarsal Osteotomy (DMMO) technique is gaining popularity mainly due to postoperative stiffness related to other techniques [**11**]. DMMO is an extra capsular cervical osteotomy of the lesser metatarsals that can elevate and shorten the ray; it consists in a dynamic correction with the adjustment displacement dictated by the progressive weight bearing, due to the unfixed bones. The osteotomy is performed from distal dorsal to plantar proximal trying to reach a 45° angle. Other pathologies that can be addressed by percutaneous surgery are deformities of other toes as Taylor’s bunionette (fifth toe protuberance), claw toes and mallet toes – these being some of the best indications for minimally invasive techniques. Because usually, both bony and soft tissue gestures are needed, minimization of operative morbidity is a constant request.

**Fig. 2:**
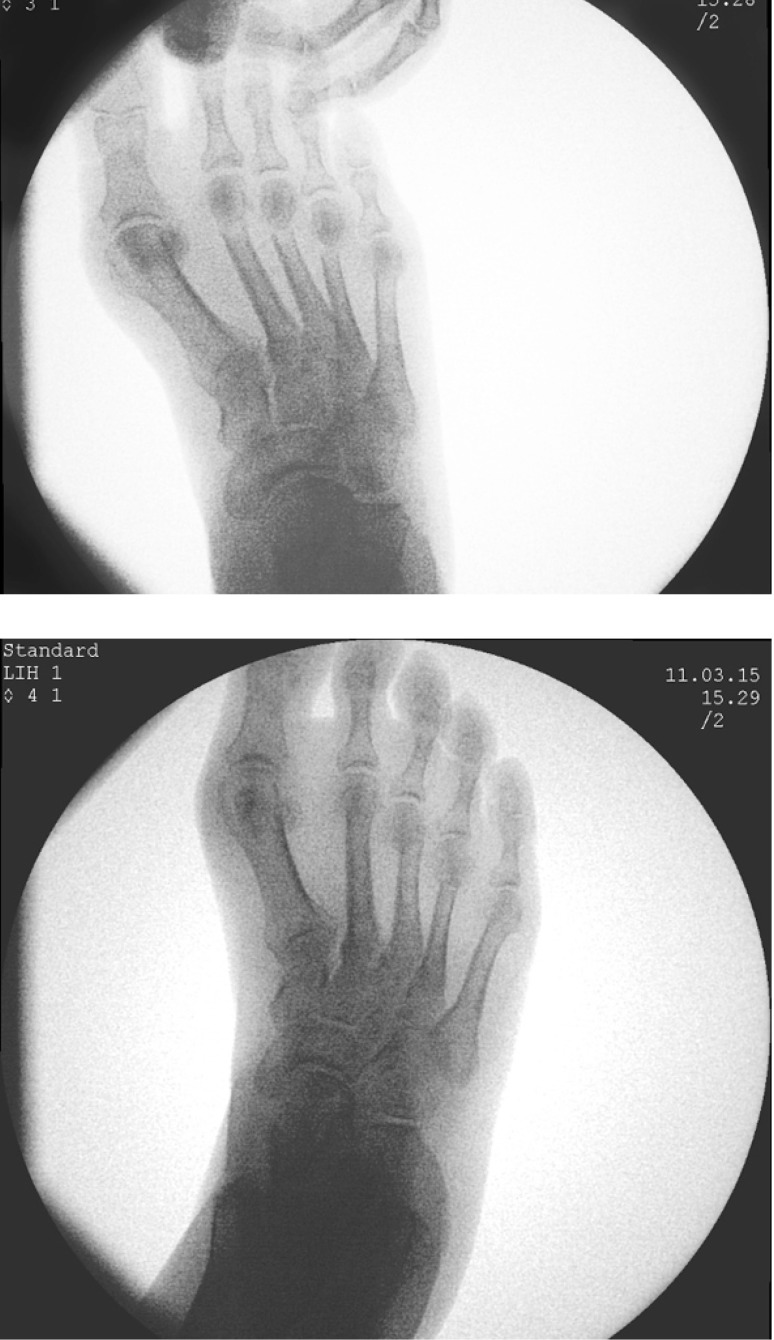
Traction and compression forces are applied along the axis of the toe for mobilization of the osteotomy and efficiency control

Arthrodeses are more advanced percutaneous procedures and can be performed by more experienced surgeons. Wedged burrs help resect the articular surfaces with the possibility to correct the desired axis; some degree of arthrolysis is always performed. One application we used is percutaneous bone exostosis shaving as an alternative procedure for residual midfoot plantar bone prominence after the reconstruction of the Charcot midfoot deformity. Percutaneous tenotomy is another effective and safe method of treating toe ulcers in neuropathic patients.

## Method

All the percutaneous techniques require specific equipment: small special blades (that make 2-3 mm in length skin incisions, can release capsule and ligaments and make tenotomies) and a special handle called beaver. The second step implies the use of specific rasps that create the working space (safe space around the bone allowing practicing osteotomies without affecting surrounding soft tissue structures) and remove bony debris. The bony procedure uses high speed burrs (described as wedge and straight Shannon) of variable width and length (usually 2 by 20 mm) for performing resection and osteotomies connected to a motor with a handheld device, like a pen, controlled by a foot pedal – allowing a controlled rotation (less than 10000 rpm). All the interventions are performed under fluoroscopy guidance by using a digitally amplified C-arm (easy to handle and less radiant).

**Fig. 3:**
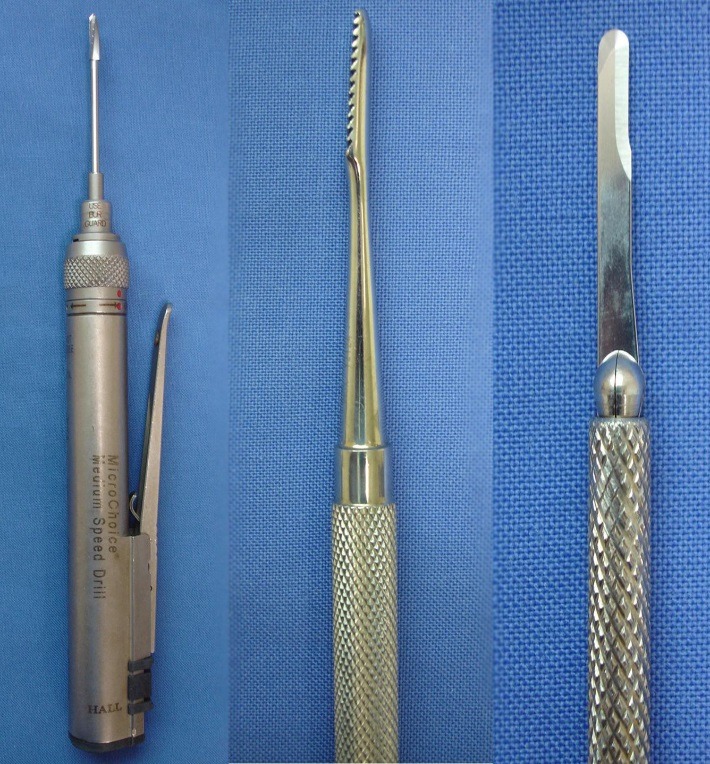
Specialized tools: from left to right – high speed burr, rasp and beaver blade

The surgeon must learn to use these specific tools, particularly the motor, because of important differences from those used in conventional surgery. Our observations are related to the specific tactile sensations associated with the percutaneous approach, various steps of the procedure, requiring various forces applied to the bone with the burr. One important step, we have mentioned earlier, is the release of the soft tissues, a crucial step in ensuring reliability and reproducibility that can be gained only through experience.

As always in percutaneous surgery, post-operative dressing is essential because it keeps the corrections in place– the technique of strapping uses bandage and gauze pads to stabilize the operated joints.

**Fig. 4:**
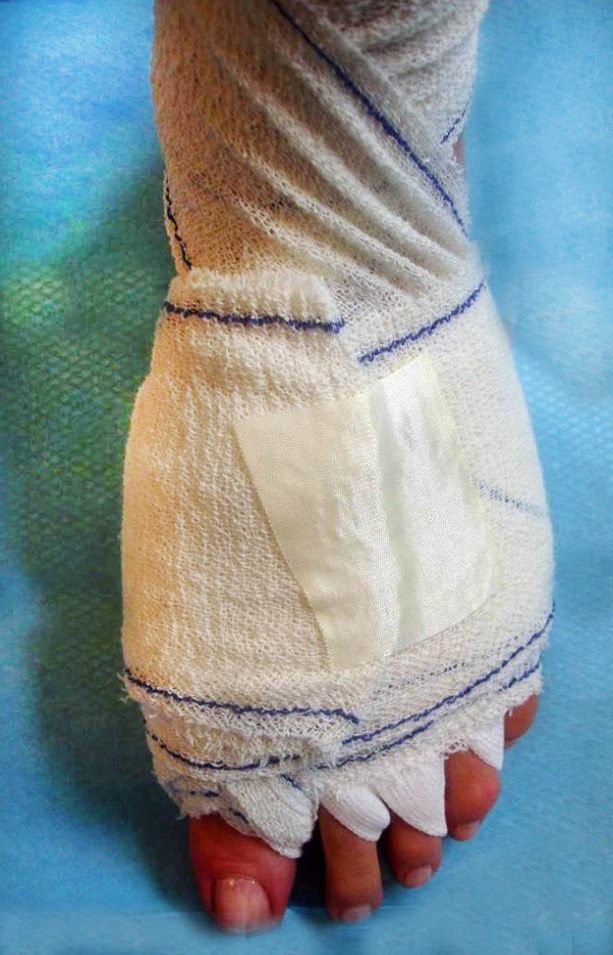
Strapping technique for postoperative dressing

## Discussion

One important conclusion retrieved from a literature review is to avoid operating for cosmetic reasons. The theoretical advantages of percutaneous osteotomies are: lower risk of stiffness (because of the limited scar and the extraarticular metatarsal osteotomy), lower risk of nonunion (because of the presence of hematoma at the osteotomy), lower risk of deep infection. Improper technique may lead to complications, including thermal injury to skin, delayed- or malunion, neurovascular damages.

Even though preliminary results are encouraging, a systematic review by Maffulli et al. revealed a lack of sufficient data to determine recommendations regarding the use of minimally invasive surgery for hallux valgus correction [**12**].

Another observation suggests that the technique is less aggressive to the patient because of the small size of scars, in that it takes place during a one-day surgery, and often under regional anesthesia. Nevertheless, it can be just as dangerous as open surgery or more in non-experienced hands [**13**,**14**].

The first danger is the difficulty of achieving these techniques, which are very attractive in appearance [**15**]. In fact, percutaneous techniques are dangerous because the tools are aggressive, and used without direct vision. This is a very different action than that accomplished in conventional orthopedics, its acquiring being imperative through instructional courses. This is the reason why several years of basic and advanced training for percutaneous surgery are organized by specialty societies.

The second danger is the erroneous application of the patient’s clinical analysis: there is nothing to respond to a purely aesthetic demand, but to make a correct choice it must be connected to pain, and difficulty of wearing shoes. As mentioned, severe hallux valgus (MTP >20 degrees) limits the efficiency of the percutaneous technique – in fact the lateral translation of the metatarsal head is directly related to the width of the head in order to obtain a good bone contact. In a recent paper, Vernois analyzed 341 patients, with emphasis on patient satisfaction and radiologic results. At more than one year of follow up (up to 3 years), patients reported 95% of good results, with only 7 cases requiring additional surgery. At 3 months, all osteotomies healed. The mean correction obtained for MTP was of 26.4 degrees and for IMA of 9 degrees, with a conclusion of a stable and effective surgical technique [**16**]. With experience, even cases with hallux valgus deformity of up to 40 degrees, even with modification of the distal metatarsal articular angle (DMAA up to 20 degrees) or with mild degenerative changes, may be approached with percutaneous osteotomies. 

Hallux valgus surgery always begins with a percutaneous milling exostosis, by using the burr under permanent control of fluoroscopy. The next step is the percutaneous osteotomy of first metatarsal neck (M1), in a precise direction to reduce the metatarsus varus and properly orient the articular surface of the head M1. The evolution of the technique started with a modification of the Reverdin osteotomy by Stephen Isham who performed it by an oblique medial closing wedge while keeping the medial hinge and combining it with the percutaneous section of the adductor hallucis and external release of the first MP joint [**17**]. As mentioned, one other associated osteotomy is an Akin type, focused on the first phalanx of the hallux to balance the tension in the first ray. Due to the association of the strapping, osteotomies are maintained open, which is important because of the absence of fixation. In the 8th day, the patient is usually reviewed, and custom manufactured silicone brace are applied, allowing bone consolidation in good position, for the next 3 weeks. The full support is permitted, on the entire sole of the foot, with a hard-soled specialized shoe. Apparently, the construct was sufficiently stable not to require additional osteosynthesis [**18**]. Literature lacks papers reviewing this technique, which was popular in the United States, but Kadakia described an unacceptable rate of complications, specifically osteonecrosis, nonunion, malunion, and recurrence [**19**]. The next step of development associated both European and American techniques resulting in the so-called SERI [**20**] - Simple, Efficient, Rapid and Inexpensive. Publications revealed good clinical results with no significant complications even at 10-year follow-up [**21**]. Concerns about these procedures are related to the same instability issues because the osteotomy at the level of the neck, is immobilized by a single K-wire rather than rigidly internally fixed. Independent analyses of this technique failed to reproduce the good clinical results, even with a second K-wire to transfix the osteotomy [**22**]. These problems led Joel Vernois and David Redfern to develop a new technique based on percutaneous techniques but internally fixed with compression screws – the above-described MICA technique [**23**]. Potential advantages are the biomechanical stability of the osteotomy by the rigid internal fixation associated with an extracapsular technique that preserves the soft tissue envelope. Edema and phlebitis are the most encountered early complications – around 1% [**24**]. De Prado quotes portal burns to be around 3%. Recurrence of the hallux valgus, pain and rigidity were also present in 3 to 4% of the cases.

Metatarsalgia is treated with percutaneous oblique osteotomy, so that the metatarsal heads adjust by elevation, and remove the pain. Second ray syndrome is the most frequent because of the isolation of the anatomical basis of M2 at the Lisfranc articulation [**25**]. The association of the M2 osteotomy with hallux valgus surgery is recommended, over the association of M3 and M4, to respect the harmonious anterior arch of the forefoot but always related to clinical symptoms in the area. Otherwise, it quickly exposes to a charge transfer, and rapid onset of pain in lateral metatarsal heads. This technique apparently simplifies the postoperative follow-up of open surgery represented by classical Weil osteotomy. However, Henry et al. observed a longer persistence of edema in the postoperative period [**11**]. The technique is also contraindicated in dislocations of distal phalanges, where only the Weil osteotomy may be reliable. Another observation is related to the postoperative radiographic controls with the presence of large bone callus. It is not a hypertrophic nonunion; the modification fades in a few months. Portal burn risk was the main complication we encountered; an exchange of the motor utilized with more control over the rotational speed led to less skin damage. Another complication is related to the under- or over-correction of the metatarsals due to the free adjustment in the postoperative interval – this was not a very important element in comparison with the good clinical results (also confirmed by less postoperative stiffness and pain in comparative studies published in literature – [**26**]).

**Table 1:**
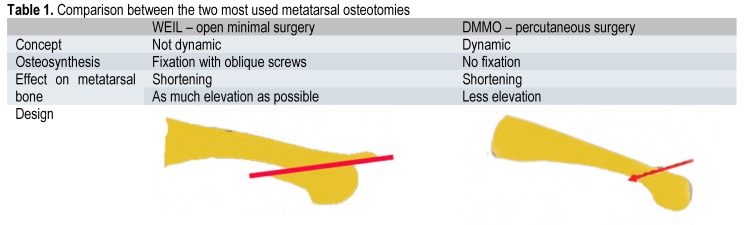
Comparison between the two most used metatarsal osteotomies

**Fig. 5:**
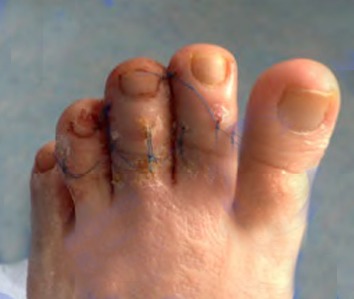
Postoperative control after claw toes minimally invasive surgery; bridging skin sutures can be a valuable surgical artifice

An important observation is related to the permanent need of soft-tissue adjustment associated with every intervention (open or minimally invasive) – from the first step of surgical access by using the special elevator to the final releases which allow the bone fragments to dynamically align. Sometimes, even at the time of the surgical closure, the wound is left without stitches to avoid hematoma; another option is to use bridging sutures to keep the deviation reduced (**Fig. 5**).

Post-operative follow-up is very important. Besides recommendations to keep the operated foot suspended as much time as possible, the dressings are renewed as frequently as needed and as much as possible under the supervision of the surgeon. With most of these techniques, the patients are allowed to weight bear as tolerated, controlling edema and pain and by using specialized shoes at least for 3 weeks.

## Conclusions

Even if reduced, literature data prove that percutaneous foot surgery can be an applicable procedure which provides comparable clinical results to those reported with conventional open techniques. Some surgeons see percutaneous surgery as a consumer, an up-to-the-minute accessory, rather than a surgical procedure. The gap between fiction and reality may be related to serious postoperative consequences – with some techniques having as much as 30% complication rate [**27**]. The inability of the orthopedic surgeon to correct the false beliefs and benefits is a formal obstacle to surgery. Caution is also needed when the patient does not understand the evolving nature of the foot pathology, the request of physical therapy and orthopedic support. Foot surgery must be an orthopaedic subspeciality, where surgeons must pass special cadaveric training before beginning these procedures.

The outpatient care, the minimal size of scars, association of regional anesthesia can induce an apparent easiness on the part of surgeons, not to forget that this surgery can be relied on heavy potential complications. In the post-operative period, time is needed to gain expertise with the specific dressings and with the radiological and clinical evolution. Before starting to perform percutaneous surgery, the surgeon must follow one or more theoretical and practical cadaver courses in order to gain hands-on familiarity with the specific tools. In our current practice, we integrate the percutaneous techniques with open surgery in order to provide the best treatment option according to the disorder and patient expectations.
